# Prediction of outcomes by early treatment responses in childhood T-cell acute lymphoblastic leukemia: a retrospective study in China

**DOI:** 10.1186/s12887-015-0390-z

**Published:** 2015-07-15

**Authors:** Wei Wei, Xiaojuan Chen, Yao Zou, Lixian Chang, Wenbin An, Yang Wan, Tianfeng Liu, Wenyu Yang, Yumei Chen, Ye Guo, Xiaofan Zhu

**Affiliations:** Department of Pediatric, Institute of Hematology and Blood Diseases Hospital, Chinese Academy of Medical Sciences and Peking Union Medical College, 288 Nanjing Road, Tianjin, 300020 Peoples Republic of China

**Keywords:** Childhood, T-cell acute lymphoblastic leukemia, Prednisone response, Minimal residual disease, Prognosis

## Abstract

**Background:**

Early treatment responses are important prognostic factors in childhood T-cell acute lymphoblastic leukemia (T-ALL) patients. The predictive values of early treatment responses in Chinese childhood T-ALL patients were still unknown.

**Methods:**

From January 2003 to December 2012, 74 consecutive patients aged ≤15 years with newly diagnosed T-ALL were treated with BCH-2003 protocol or CCLG-2008 protocol in the Department of Pediatric, Institute of Hematology and Blood Diseases Hospital in China. Predictive values of early treatment responses, including prednisone response, bone marrow morphology at day 15 and day 33 during induction chemotherapy, and minimal residual disease (MRD) monitored by flow cytometry after induction therapy (time point 1, TP1) and before consolidation therapy (time point 2, TP2), were analyzed.

**Results:**

The 5-year event free survival (EFS) and overall survival (OS) rates for these patients were 62.5 % (SE, 6.4) and 62.7 % (SE, 6.6), respectively. Prednisone poor responder was strongly associated with increased chance of induction failure (14.8 %) and decreased survival rate (5 year EFS rate, 51.1 % (SE, 10.5)). Patients with ≥25 % blast cells in bone marrow at day 15 were more likely to have an inferior outcome. 93.2 % of the T-ALL patients achieved complete remission at day 33 while patients with resistant disease all died of disease progression. MRD ≥10^−2^ at TP1 or MRD ≥10^−3^ at TP2 was significantly related to dismal prognosis. Risk groups classified by MRD at two time points could stratify patients into different groups: 29.0 % of the patients were MRD standard risk (MRD < 10^−4^ at both time points) with 3-year EFS rate of 100 %, 29.0 % were MRD high risk (MRD ≥10^−2^ at TP1 or MRD ≥10^−2^ at TP2) with 3-year EFS rate of 55.6 % (SE, 16.6) , and the rest of patients were defined as MRD intermediate risk with 3-year EFS rate of 85.7 % (SE, 13.2).

**Conclusion:**

Our study demonstrated that MRD was the most powerful predictor of treatment outcome in childhood T-ALL patients and conventional morphological assessments of treatment response still played important roles in predicting treatment outcome and tailoring treatment intensity especially in countries with inadequate skills or financial resources for MRD monitoring.

**Electronic supplementary material:**

The online version of this article (doi:10.1186/s12887-015-0390-z) contains supplementary material, which is available to authorized users.

## Background

Acute lymphoblastic leukemia (ALL) with T-cell immunophenotype accounting for approximately 15 % of the childhood ALL patients was considered to be unfavorable until more intensive chemotherapy had be applied in the last two decades [1–6]. Childhood T-cell acute lymphoblastic leukemia (T-ALL) patients are more likely to be male, older than 9 years and present with high white blood cell (WBC) count, mediastinal mass and central nervous system leukemia [1, 2, 6].

Early in vivo responses are known to be powerful predictors of treatment outcome in childhood ALL [7–11]. Prednisone response (PR) was found to be related to treatment outcome by the Berlin-Frankfurt-Munster (BFM) study in 1983 [7] and since then the predictive value of PR was confirmed in many studies [12–15]. T-ALL patients are more likely to have a worse steroid response than B-ALL patients [8, 10, 16]. Bone marrow morphology at day 15 during induction therapy is a well-established predictive factor and patients with ≥25 % blast cells in bone marrow usually have an inferior survival [9, 13, 17, 18]. During the last two decades, minimal residual disease (MRD) in childhood ALL had been proved to be a remarkable predictive factor and already become an integral part of risk stratifications in many long established leukemia groups [19–24]. The most widely applicable MRD technique is polymerase chain reaction (PCR) analysis of T-cell receptor (TCR) and clone-specific immunoglobulin gene arrangements [25–27]. Although less standardized than molecular detection of MRD, flow cytometry (FCM) is faster, cheaper and more applicable [18, 22, 28–31]. Patients’ prognosis and quality of life were further improved by individualized treatment. The majority of MRD studies were based on B-ALL, whereas MRD studies in T-ALL were scarce. A slower clearance of leukemia cells was found in T-ALL patients and MRD risk group classified by MRD levels at the end of induction and before consolidation therapy was identified to be the most powerful independent prognostic factor in T-ALL patients [32, 33].

In this study, the clinical features and early treatment responses of Chinese pediatric T-ALL patients were summarized. The predictive values of early treatment responses, including prednisone response, bone marrow morphology at day 15 and day 33 during induction therapy, MRD levels after induction and before consolidation therapy, and their correlations were analyzed. Prednisone response, bone marrow morphology at day 33, and MRD were identified to be powerful prognostic factors in our T-ALL patients. This is the first time that the predictive values of early treatment responses especially MRD levels were explored in Chinese pediatric T-ALL patients.

## Methods

### Patients and treatment protocols

From January 2003 to December 2012, 74 consecutive patients aged 15 years or younger with newly diagnosed T-ALL were enrolled in the Department of Pediatric, Institute of Hematology and Blood Diseases Hospital, Peking Union Medical College. The diagnosis of ALL was based on morphologic, cytochemical, and immunophenotypic criteria. T-cell lineage was established based on the European Group for the Immunological Characterization of Leukemias criteria (EGIL). 27 patients and 47 patients were treated with BCH-2003 protocol (used between January 2003 and March 2008) and CCLG-2008 protocol (used after April 2008), respectively. Patients were stratified into intermediate risk (IR) and high risk (HR) groups according to cytogenetic aberration, prednisone response, bone marrow morphology at day 15 and 33, and MRD levels (The details of stratification criteria and treatment protocols were described in Additional file 1: Table S1, S2, S3). One patient treated with BCG-2003 and one treated with CCLG-2008 protocol received allogeneic hematopoietic stem cell transplantation (allo-HSCT). Written informed consent from the parents or guardians of the study participants were obtained in accordance to the Declaration of Helsinki before initiation of treatments, and the protocols were approved by the ethics committee of Institute of Hematology and Blood Diseases Hospital.

### Flow cytometric assessment of minimal residual disease

MRD was included to stratify risk group in CCLG-2008 protocol. Thus, MRD levels were monitored in patients treated with CCLG-2008 protocol. Bone marrow aspirates were collected in preservative-free heparin at the end of remission induction (on day 33) and before consolidation therapy (in week 12). Leukemia-associated immunophenotypes were determined by multivariable flow cytometry and multiple marker combinations (CD7/CD45/CD33/CD34/CD117/CD10/CD2/cCD3/TDT and CD7/CD45/CD3/CD4/CD8/CD99/CD5/CD16/CD56) were performed in the Department of Pathology in our hospital.

### Early response and relapse criteria

Prednisone response (PR) was defined by the absolute number of leukemia blasts in the peripheral blood after seven days of prednisone treatment and one intrathecal (IT) dose of methotrexate. The number of peripheral blasts of prednisone good responder (PGR) was <1000/ul, whereas the value of prednisone poor responder (PPR) was ≥1000/ul. BM morphology was evaluated at day 15 during induction therapy and BM statuses were defined as M1 (lymphoblasts < 5 %), M2 (≥5 % and <25 %) and M3 (≥25 %). Complete remission (CR) was defined as normal BM cellularity with <5 % undifferentiated cells at day 33 of induction therapy, absence of leukemia blasts in peripheral blood and CSF, and no extramedullary infiltration. MRD were assessed at the end of induction treatment (day 33, time point 1, TP1) and in week 12 before consolidation therapy (time point 2, TP2) by flow cytometry. Relapse was defined as recurrence of ≥25 % lymphoblasts in bone marrow or local leukemia infiltration sites.

### Statistics

December 31, 2012 was chosen as the reference date for collection of data. Chi-square test was used for comparison of binary variables, and Mann–Whitney U test was used for comparison of continuous variables. Outcome events were induction failure, induction death, relapse, death during remission, and secondary malignancy. Event free survival (EFS) was defined as the time from diagnosis to the date of last follow in CR or first event. Induction failure or induction death were considered to be event at time zero. Overall survival (OS) was measured from the date of diagnosis to the time of death from any cause. Patients lost to follow up were censored at the time of their withdrawal. Distributions of EFS and OS rates were estimated by the Kaplan-Meier method [34] and differences were compared with two-sided log rank test [35]. The Cox proportional-hazards model was used for multivariate analyses of prognostic factors [36]. Estimated hazard ratios were reported as relative risks with 95 % confidence intervals. All *P* values were two-sided and *P* < 0.05 was considered statistically significant. Statistical analyses were performed using SPSS 17.0 software.

## Results

### Patient characteristics

Presenting clinical features of the 74 T-ALL patients and the outcomes associated with clinical characteristics were summarized in Table 1. 57 (77.0 %) patients were male and 17 (33.0 %) patients were female. Ages ranged from 1 to 15 years with a median age of 9 years. 45 (60.8 %) patients presented with initial white blood cell (WBC) count ≥100 × 10^9^/L. 29 (40.3 %) patients were classified as the intermediate risk (IR) group and 43 (59.7 %) patients were in the high risk (HR) group according to the risk stratifications. 27 (36.5 %) patients followed BCH-2003 protocol and 47 (63.5 %) were treated with CCLG-2008 protocol. The median follow-up time of the two protocols were 73 and 19 months, respectively. There were no significant differences in the distributions of age, sex, leukocyte count, risk group, karyotype and early treatment responses between BCH-2003 protocol and CCLG-2008 protocol, but patients with CNS3 status were more in BCH-2003 protocol and more patients with mediastinal mass were found in CCLG-2008 protocol (Additional file 1: Table S4).

**Table 1:** Clinical characteristics and outcomes

Variables	No. (%)	5-year EFS (SE)	*P* value
Total	74	62.5 (6.4)	
Treatment protocol			
BCH-2003	27 (36.5)	61.7 (10.8)	0.274
CCLG-2008	47 (63.5)	55.6 (9.6)	
Gender			
Male	57 (77.0)	60.7 (7.1)	0.461
Female	17 (33.0)	65.9 (16.5)	
Age (years)			
1-10	43 (58.1)	55.8 (8.8)	0.47
≥10	31 (41.9)	73.9 (8.0)	
Initial WBC (×10^9^/L)			
<100	29 (39.2)	68.2 (11.6)	0.076
≥100	45 (60.8)	58.0 (7.8)	
CNS involvement			
CNS1/2	68 (91.9)	64.9 (6.7)	0.034
CNS3	6 (8.1)	33.3 (19.2)	
Mediastinal mass			
Present	28 *(*40.6)	68.8 (9.6)	0.805
Absent	41 (59.4)	59.7 (8.3)	
Not known	5		
MLL rearrangement			
Present	3 (6.1)	100.0	0.217
Absent	46 (93.9)	57.0 (10.6)	
Not known	25		
karyotype			
Normal	31 (56.4)	67.5 (9.2)	0.163
Structure abnormal	17 (30.9)	82.4 (9.2)	
Numerical abnormal	7 (12.7)	28.6 (22.3)	
Failure or Missing	19		
Risk group			
IR	29 (40.3)	79.0 (10.3)	0.009
HR	43 (59.7)	54.8 (8.2)	
Not known	2		
SIL-TAL1 translocation		3-year EFS (SE)	
Present	8 (19.0)	100.0	0.102
Absent	34 (81.0)	51.7 (12.3)	
Not known	32		

*WBC* White blood cell; *IR* Intermediate risk; *HR* High risk; *SE* Standard error; *CNS* Central nervous system

By Kaplan-Meier method

### Treatment outcome

**Fig. 1:**
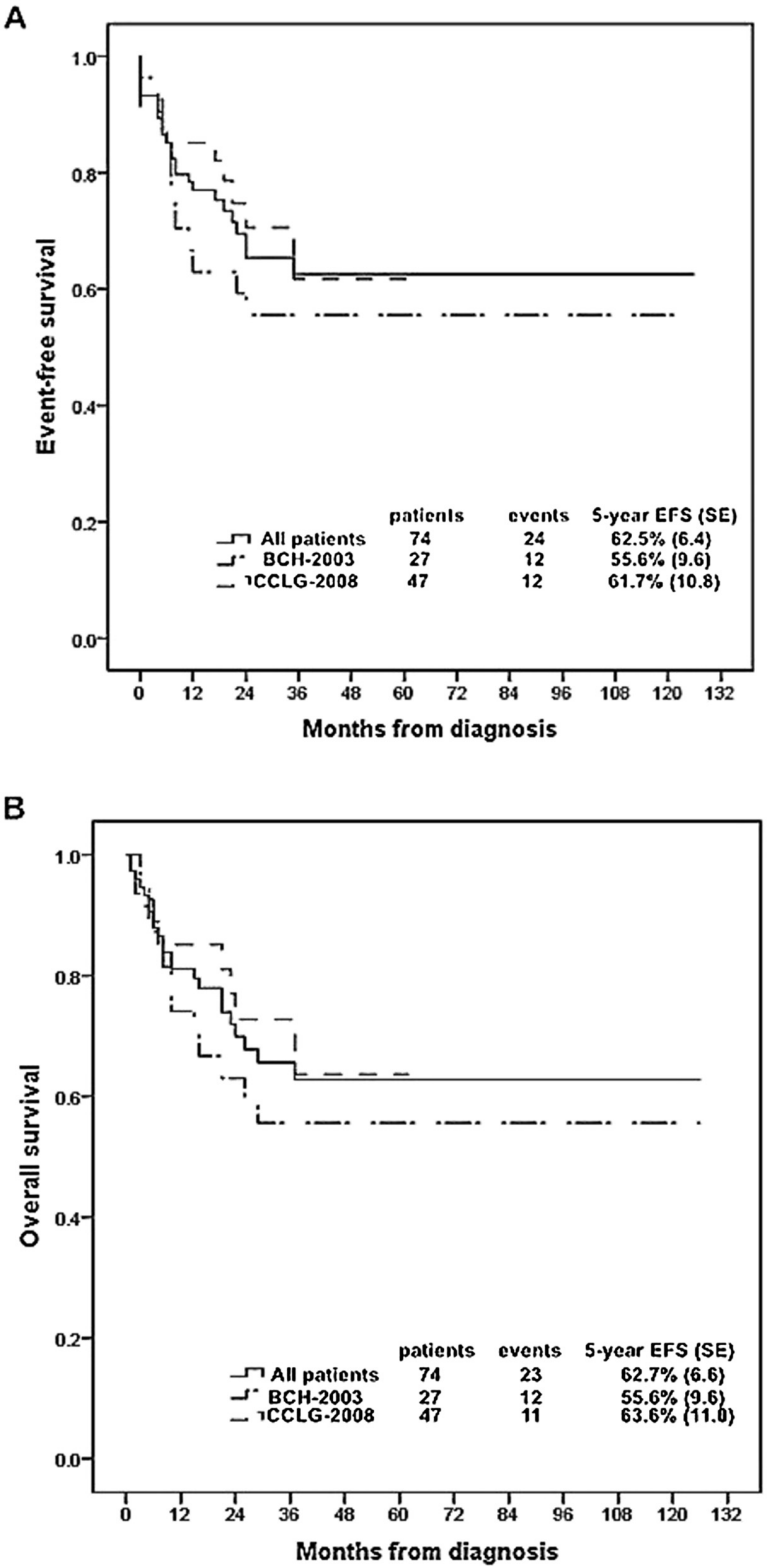
**a** Event-free survival (EFS) rates and **b** Overall survival (OS) rates for all the patients and patients classified by treatment protocols (BCH-2003 protocol or CCLG-2008 protocol )

The 5-year EFS and OS rates for all patients were 62.5 % (SE, 6.4) and 62.7 % (SE, 6.6), respectively, with a median follow-up of 22 months (Fig. 1). Complete remission (CR) could be assessed in 72 patients on day 33 of induction therapy. 69 (93.2 %) patients achieved CR and 5 patients failed. Of the 5 patients, 3 patients suffered induction resistance and 2 died during induction chemotherapy. None of the patients with induction resistance achieved CR after the intensified re-induction therapy and all of them died of disease progression. 15 (20.3 %) patients relapsed in bone marrow isolated (*n* = 11) or combined CNS (*n* = 4) or testis (1 patients relapsed at BM combined both CNS and testis). 10 (66.7 %) patients relapsed within 18 months and the others relapsed between 18 months and 36 months. Other events were induction failure (*n* = 3), induction death (*n* = 2), and death in remission (*n* = 4).

The 5-year EFS rates for patients treated with BCH-2003 and CCLG-2008 protocols were 55.6 % (SE, 9.6) and 61.7 % (SE, 10.8), respectively (*P* = 0.274), and the 5-year OS rates were 55.6 % (SE, 9.6) and 63.6 % (SE, 11.0), respectively (*P* = 0.283) (Fig. 1). 6 patients had central nervous system (CNS) involvement at the time of diagnosis and their 5-year EFS rate was much lower than that of the patients who were CNS1 or CNS2 status, 33.3 % (SE, 19.2) and 64.9 % (SE, 6.7), respectively (*P* = 0.034). The 5-year EFS rates were significantly different between the IR and HR groups (79.0 % (SE, 10.3) VS 54.8 % (SE, 8.2), respectively; *P* = 0.009). Patients who had SIL-TAL1 translocation seemed to have a better outcome with the 3-year EFS rate of 100 %, but without significance due to the small number of patients (*P* = 0.102). As shown in Table 1, no significant differences were observed in EFS rates based on: age at diagnosis (*P* = 0.47), sex (*P* = 0.461), presence of mediastinal mass (*P* = 0.85), MLL rearrangement (*P* = 0.217) or karyotype (*P* = 0.163).

### Prednisone response

**Fig. 2:**
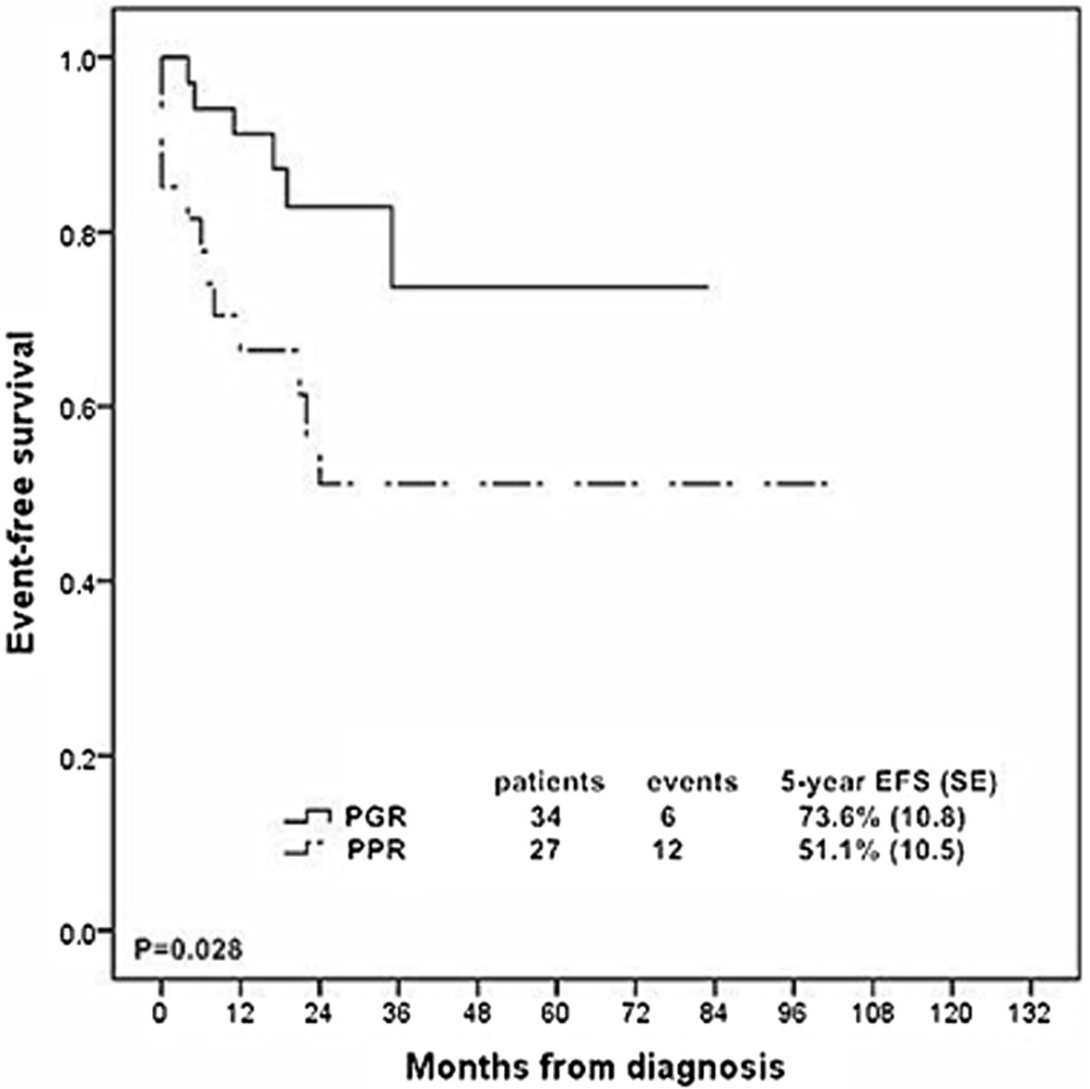
Kaplan-Meier estimate of event-free survival according to prednisone response in 61 T-ALL patients. PGR: prednisone good responder; PPR: prednisone poor responder; SE, standard error

Prednisone response could be evaluated in 61 patients. Of those patients, 34 (55.7 %) patients were defined as prednisone good responder (PGR), while 27 (44.3 %) were classified as prednisone poor responder (PPR). The relationships between PR and clinical features were analyzed and patients with initial WBC ≥ 100 × 10^9^/L were more likely to respond poorly to prednisone (*P* = 0.033, Additional file 1: Table S5). PPR patients had a significant lower 5-year EFS rate than PGR patients. The 5-year EFS rate was 51.1 % (SE, 10.5) for PPR patients compared to 73.6 % (SE, 10.8) for PGR patients (*P* = 0.028, Fig. 2).

### Bone marrow morphology at day 15

**Fig. 3:**
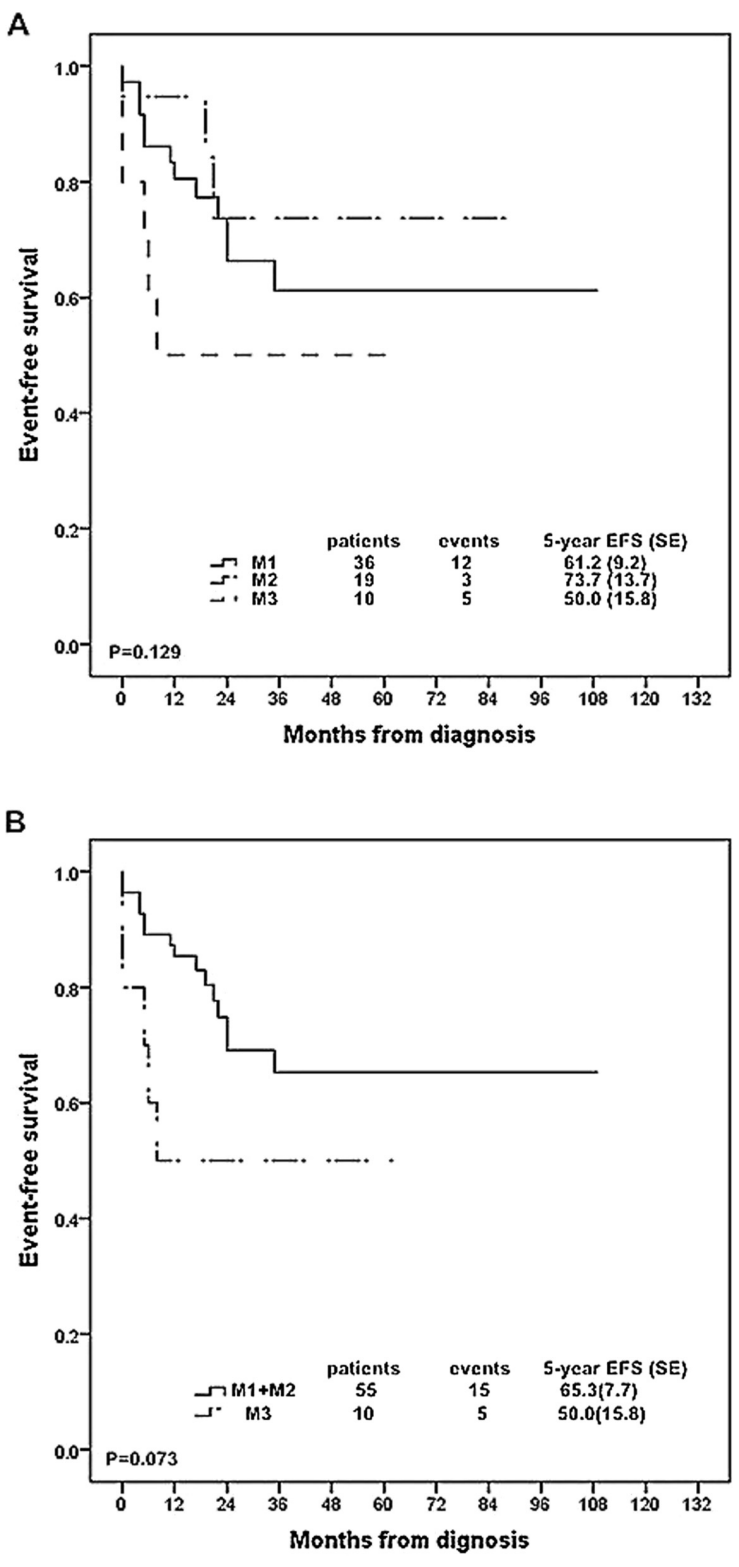
Kaplan-Meier estimate of event-free survival according to bone marrow morphology at day 15 during induction chemotherapy in 65 T-ALL patients. **a** Patients classified into three groups: M1 (bone marrow blast <5 %), M2 (bone marrow blast 5 % and <25 %), and M3 (bone marrow blast ≥25 %). **b** Patients classified into two groups: M1 + M2 (bone marrow blast <25 %) and M3 (bone marrow blast ≥25 %). SE, standard error

Bone marrow smears at day 15 of induction therapy were eligible for evaluation in 65 patients. 36 (55.4 %) patients were defined as M1 status, 19 (29.2 %) patients were classified as M2 status and 10 (15.4 %) patients were defined as M3 status. The 5-year EFS rates were 61.2 % (SE, 9.2), 73.7 % (SE, 13.7) and 50.0 % (SE, 15.8) for the patients with M1, M2 and M3 status, respectively (*P* = 0.129, Fig. 3a). M3 status at day 15 is internationally recognized as a poor prognostic factor and there was no difference in treatment outcome between M1 and M2 patients in our study. Thus, we combined M1 and M2 patients into one group to compare with M3 patients. The 5-year EFS rate for M3 patients was lower than that for M1/2 patients with borderline significance (50 % (SE, 15.8) VS 65.3 % (SE, 7.7), *P* = 0.073, Fig. 3b). The relationships between clinical features and BM status at day 15 were also analyzed and no significant correlation was found (Additional file 1: Table S6).

### MRD at day 33 and in week 12

33 and 32 patients were evaluable for MRD analysis at day 33 (TP1) and in week 12 (TP2), respectively. All the patients were treated with CCLG-2008 protocol. At first, we explored the cutoff values of MRD levels at each time point. Patients eligible for MRD analysis were divided into subgroups according to their MRD levels at TP1 or TP2: MRD < 10^−4^ and ≥10^−4^, MRD <10^−3^and ≥10^−3^, MRD < 10^−2^/≥10^−2^. Numbers of patients and their 3-year EFS rates for these subgroups were assessed and showed in Table 2. Patients with MRD ≥10^−2^ at any time point had the worst outcome (3-year EFS rate of 33.3 % (SE, 27.2 %) at either time point). At TP2, patients could also be classified into two groups by MRD level of 10^−3^. 23 (71.9 %) patients with MRD < 10^−3^ had an excellent 3-year EFS rate compared with patients with MRD ≥10^−3^( 87.5 % (SE, 11.7) and 55.6 % (SE, 16.6), respectively).

**Table 2:** Distribution of MRD levels at two time points and comparison of event-free survival in patients classified by MRD levels

Time point	MRD cut-off	N (%) of patients	3-year EFS (%, SE)	Long-rank test
	values			
Time point 1	<10^−4^/≥10^−4^	16 (48.5)/17 (51.5)	80 (17.9)/73.2 (11.9)	*P* = 0.337
	<10^−3^/≥10^−3^	27 (81.8)/6 (18.2)	77.2 (12.7)/66.7 (19.2)	*P* = 0.243
	<10^−2^/≥10^−2^	30 (90.9)/3 (9.1)	80.8 (10.8)/33.3 (27.2)	*P* = 0.006
Time point 2	<10^−4^/≥10^−4^	14 (43.8)/18 (56.2)	100/69.1 (11.9)	*P* = 0.059
	<10^−3^/≥10^−3^	23 (71.9)/9 (28.1)	87.5 (11.7)/55.6 (16.6)	*P* = 0.004
	<10^−2^/≥10^−2^	29 (90.6)/3 (9.4)	83.8 (9.8)/33.3 (27.2)	*P* = 0.002

*SE* Standard error

**Fig. 4:**
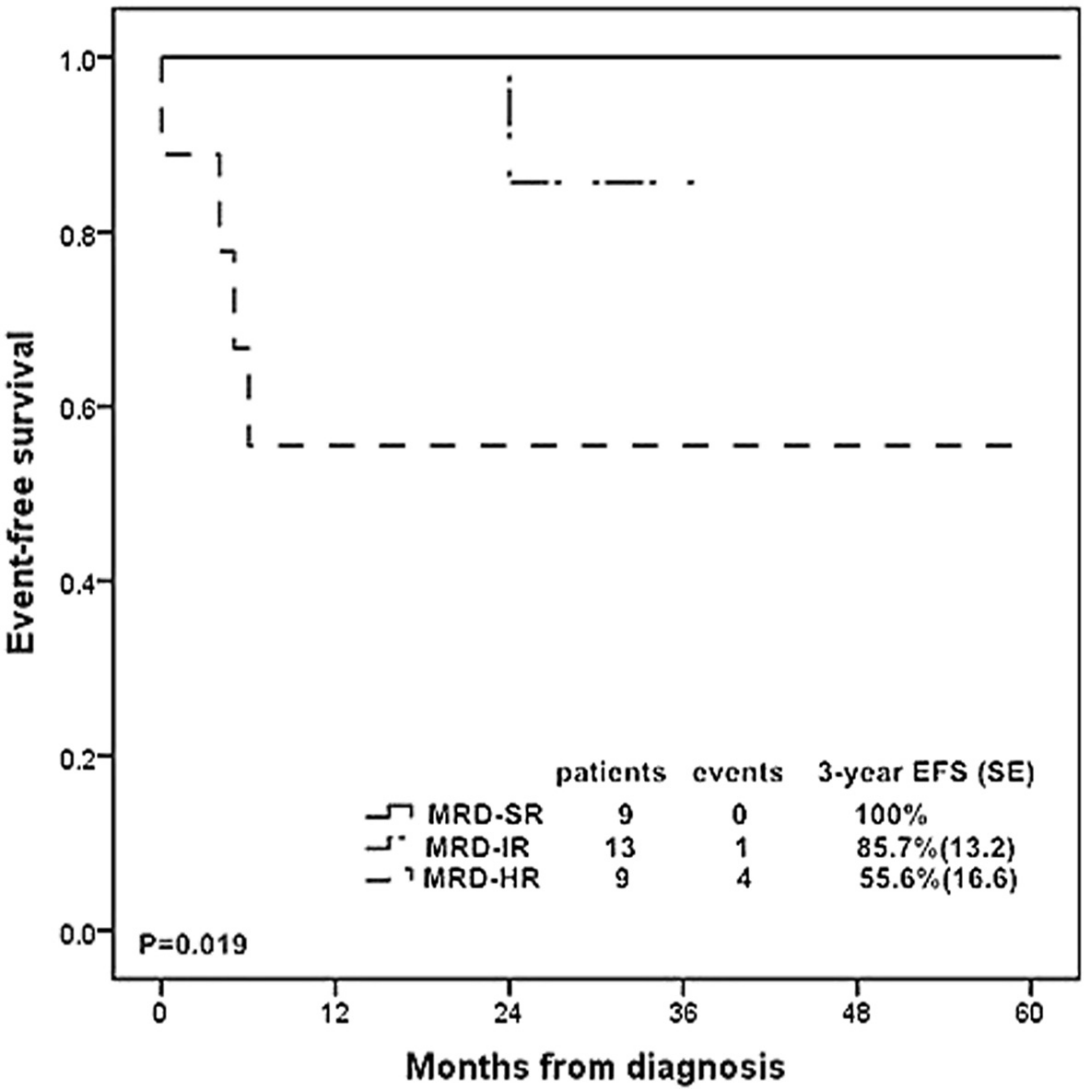
Kaplan-Meier estimate of event-free survival according to minimal residual disease (MRD) risk groups. Patients with MRD < 10^−4^ at TP1 and TP2 were stratified as the standard-risk group (MRD-SR); MRD ≥ 10^−2^ at TP1 or MRD ≥ 10^−3^ at TP2 as high-risk group (MRD-HR); the rest as the intermediate risk group (MRD-IR)

According to the above analysis of MRD levels at TP1 and TP2, we subsequently stratified the patients into three MRD risk groups: 9 ( 29.0 %) patients with MRD <10^−4^ at both time points were defined as standard risk (MRD-SR); 9 (29.0 %) patients with MRD ≥10^−2^ at TP1 or ≥10^−3^ at TP2 were at high risk group (MRD-HR) and 13 (42.0 %) patients were defined as intermediate risk (MRD-IR) group. These subgroups had distinct outcomes, with 3-year EFS rates of 100 %, 85.7 % (SE, 13.2), and 55.6 % (SE, 16.6) for MRD-SR, MRD-IR, and MRD-HR, respectively (*P* = 0.019, Fig. 4). The correlations between MRD risk groups and clinical features were analyzed and no association was observed (Additional file 1: Table S7).

### Correlations of the early treatment responses

We further analyzed if prednisone response was also a good predictor to other treatment responses. Morphological evaluation of bone marrow at day 15 was performed in 59 of the 61 patients with PR results. In PGR patients, only 3 (8.8 %) patients were defined as M3 status while 7 (28.0 %) patients out of the 25 PPR patients were M3 status (*P* = 0.019). All of the PGR patients achieved CR while the CR rate of the PPR patients was only 85.2 % (*P* = 0.034). 50 % of the PPR patients were in MRD-HR group whereas the proportion of PGR patients was only 16.7 % (*P* = 0.102). Thus, patients who responded poorly to prednisone tended to be poor responders in the late course of chemotherapy.

Age, WBC count, gender, CNS involvement, risk group, prednisone response, bone marrow at day 15 and MRD risk group were included in the multivariable analysis of event free survival. Only MRD risk group was found to be the significant independent prognostic factor (*P* = 0.032, RR = 11, 95 % CI, 1.2-100). The number of patients in the Cox regression model was limited by patients eligible for MRD risk group assessment, so if MRD risk group was omitted form the model, PPR was significantly related to the hazard of events (*P* = 0.044, RR = 2.79, 95%CI, 1.03-7.58).

## Discussion

To our knowledge, this is the first study about early treatment responses in Chinese pediatric T-ALL patients. Since last decade, the outcome of pediatric B-ALL patients has been progressively improved in China, with an overall 5-year EFS rate of approximately 85 % [37]. However, the prognosis of T-ALL patients was still not optimistic, with a 5-year EFS rate of around 65 % [37]. In our study, the 5-year EFS and OS rates for the T-ALL patients were 62.5 % (SE, 6.4) and 62.7 % (SE, 6.6), which were lower than that in western countries [25, 26, 38–41]. Only two patients in HR group underwent allo-HSCT and the rest patients in HR group were just treated with chemotherapy because of financial reason or without appropriate donors. Low percent of patients receiving bone marrow transplantation in HR group compromised our treatment outcome. The major event was relapse and most patients (66.7 %) relapsed within 18 months from diagnosis. Almost all of the patients with relapse gave up due to limited financial resource or died of disease progression because of poor treatment response. Abandonment after relapse was another reason for low survival rate compared with other studies. Thus, strategies should be made to decrease abandonment and improve the outcome of patients with relapse in China or other underdeveloped countries.

Like other pediatric T-ALL studies, the majority of our patients were male and the median age was 9 years old [1, 2, 6]. Patients with initial WBC count ≥100 × 10^9^/L accounted for 60.8 % and seemed to have a worse survival. More than half of the patients were in the HR group with 5-year EFS rate of 54.8 % (SE, 8.2) whereas the 5-year EFS rate for the IR group was much higher, 79.0 % (SE, 10.3). Of the presenting clinical features, CNS leukemia was strongly associated with poor treatment outcome. Thus, efforts to increase T-ALL survival should be focused on patients in HR group or with CNS leukemia in our future study.

Prednisone response has consistently been found to be one of the most powerful independent prognostic factors in many studies [7, 12–15] and T-ALL patients are more likely to be PPR [10, 16]. 55.7 % of our T-ALL patients were classified as PPR while the percent was only 5-15 % in B-ALL patients [8, 10, 16]. Patients with initial WBC ≥100 × 10^9^/L were at high risk of PPR (*P* = 0.033) in our study. We proved that the prognosis of PPR patients was inferior to that of the corresponding PGR patients in Chinese pediatric T-ALL patients. We further explored the correlations of PR with other treatment responses. If patients responded poorly to prednisone, they were more likely to be defined as M3 bone marrow status at day 15, underwent induction failure at day 33 and fell into the MRD-HR group than PGR patients. Thus, prednisone response is a robust predictor, inexpensive and convenient tool to predict treatment outcome and adapt treatment intensity, especially in underdeveloped countries with inadequate skills and resources for MRD monitoring.

For more than two decades, cytomorphological responses of bone marrow have been the leading strategies for risk classification [10, 13, 17, 18]. In our study, we analyzed bone marrow morphologies at day 15 and day 33 of induction therapy. At day 15, patients with M3 status had a worse outcome than M1/M2 patients with borderline significance (*P* = 0.073). The CR rate of the patients evaluated on day 33 was 93.2 % and equal to other western studies [37–40]. Patients with induction resistance all died of disease progression making induction failure the worst predictive factor in all of the early treatment response indicators.

MRD is widely applied in contemporary childhood ALL studies. In the AIEOP-BFM-ALL 2000 study, assessments of MRD at day 33 and day 78 based on immunoglobulin and TCR gene rearrangements were introduced for risk stratification [33]. In our study, MRD levels measured by FCM at day 33 and in week 12 were incorporated in risk-classification algorithms and used to adapt therapy in CCLG-2008 protocol. Thus, the numbers of patients with MRD data decreased to 33 at TP1, 32 at TP2 and 31 at both time points. Then we wanted to find out the cut-off values of MRD levels at TP1 and TP2. MRD levels ≥10^−2^ were found to be related to poor prognosis at both time points especially at day 33. In week 12, MRD at the level of 10^−3^ was considered more appropriate than 10^−2^ as it could identify more patients with dismal prognosis. According to our MRD cut-off levels, we stratified our patients into three MRD risk groups: 29.0 % of the patients were MRD-SR ( MRD < 10^−4^ at both time points), 29.0 % were MRD-HR (MRD ≥10^−2^ at TP1 or ≥10^−3^ at TP2) and 42.0 % were MRD-IR. This constitution of MRD risk groups was similar to previous MRD study in T-ALL patients [33]. The MRD-HR group displayed a remarkable worse outcome than the MRD-SR and MRD-IR groups. Cox regression analysis also showed that MRD-HR patients had a significant 11-fold increase of events compared with MRD-SR and MRD-IR patients. The reliability of our MRD analysis might be weakened by small number of patients. However, our study could still provide us with an understanding of the role of MRD in childhood T-ALL patients especially in Chinese T-ALL population and treatment protocols.

## Conclusion

Our study showed that early treatment responses were important predictors of outcome in childhood T-ALL patients. Prednisone response was still one of the most powerful predictive factors even in MRD based protocol. Thus, traditional morphologic assessments of tumor burden still play important roles in modern T-ALL treatment protocols especially in underdeveloped countries. Our study also demonstrated that MRD levels detected by FCM at two time points were significantly independent prognostic factors and MRD based stratification was superior to stratifications based on other conventional risk factors.

## Additional file

Additional file 1: Table S1. BCH-2003 chemotherapy protocol. **Table S2**. CCLG-2008 chemotherapy protocol. **Table S3**. Stratification criteria of BCH-2003 and CCLG-2008 treatment protocol. **Table S4**. Clinical characteristics of patients according to protocols. **Table S5**. Clinical characteristics of patients according to prednisone response. **Table S6**. Clinical characteristics of patients according to bone marrow response on day 15. **Table S7**. Clinical characteristics of patients according to MRD risk group. **Table S8**. Clinical characteristics of patients with or without MRD data

## Abbreviations

ALL: Acute lymphoblastic leukemia
T-ALL: T-cell acute lymphoblastic leukemia
B-ALL: B-cell acute lymphoblastic leukemia
MRD: Minimal residual disease
PR: Prednisone response
PPR: Prednisone poor responder
PGR: Prednisone good responder
TP1: Time point 1
TP2: Time point 2
SR: Standard risk
IR: Intermediate risk
HR: High risk
SE: Standard Error
WBC: White blood cell
TCR: T-cell receptor
allo-HSCT: Allogeneic hematopoietic stem cell transplantation
EFS: Event free survival
OS: Overall survival
CR: Complete remission
CNS: Central nervous system
PCR: Polymerase chain reaction
FCM: Flow cytometry
