# Supplementary material for: Prediction of outcomes by early treatment responses in childhood T-cell acute lymphoblastic leukemia: a retrospective study in China
Source: BMC Pediatr. 2015 Jul 15;15:80. doi: 10.1186/s12887-015-0390-z (PMC4502910; doi:10.1186/s12887-015-0390-z)
Supplement: Additional file 1: Table S1. — BCH-2003 chemotherapy protocol. Table S2. CCLG-2008 chemotherapy protocol. Table S3. Stratification criteria of BCH-2003 and CCLG-2008 treatment protocol. Table S4. Clinical characteristics of patients according to protocols. Table S5. Clinical characteristics of patients according to prednisone response. Table S6. Clinical characteristics of patients according to bone marrow response on day 15. Table S7. Clinical characteristics of patients according to MRD risk group. Table S8. Clinical characteristics of patients with or without MRD data [file 12887_2015_390_MOESM1_ESM.docx]

**Supplementary Table 1**. **BCH-2003 chemotherapy protocol**

| **Treatment Block** | **Intermediate-risk** | **High-risk** |
| --- | --- | --- |
| **Induction** | **VDLP (5 weeks)**  - Prednisolone 60mg/m^2^ D1-28, taper over 9 days  - L-Asparaginase 5000unit/m^2^ Day 12, 15, 18, 21, 24, 27, 30, 33  - Vincristine 1.5mg/m^2^ D8, 15, 22, 29  - Daunorubicin 30mg/m^2^ D8, 15,22,29(4x)  - IT MTX D1, Triple D15, 33 | **VDLP (5 weeks)**  - Prednisolone 60mg/m^2^ D1-28, taper over 9 days  - L-Asparaginase 5000unit/m^2^ Day 12, 15, 18, 21, 24, 27, 30, 33  - Vincristine 1.5 mg/m^2^ D8, 15, 22, 29  - Daunorubicin 30 mg/m^2^ D8, 15,22,29(4x)  - IT MTX D1, Triple D15, 33 |
| **Early Intensification** | **CAM x 2 (week 5-9)**  - Cyclophophamide 1000mg/m^2^ D36  - Ara-C 75 mg/m^2^ D38-41, 45-48  - 6MP 50 mg/m^2^ D36-49  - IT Triple D45 | **HR Blocks 1, 2, 3**  *HR Block 1*  - Dexamethasone 20mg/m^2^, D1-5  - Vincristine 1.5mg/m^2^ D1, 6  - HD-MTX 5000mg/m^2^, D1  - Cyclophosphamide 200mg/m^2^, D2-4  - Cytarabine 2000mg/m^2^ D5  - E. coli L-asparaginase 25000U/m^2^ D6, 11  - Triple IT D1  *HR Block 2*  - Dexamethasone 20mg/m^2^, D1-5  - Vindesine 3mg/m^2^ D1, 6  - HD-MTX 5000mg/m^2^, D1  -Ifosfamide 800mg/m^2^ D2-4  - E. coli L-asparaginase 25000U/m^2^ D6, 11  -Daunorubicin 30mg/m^2^, D5  - Triple IT D1  *HR Block 3*  - Dexamethasone 20mg/m^2^, D1-5  - HD-Ara-C 2000mg/m^2^ D1, 2.  -Etoposide 100 mg/m^2^ D3-5  - E. coli L-asparaginase 25000U/m^2^ D6, 11  -Triple IT D5 |
| **Consolidation** | **6-MP+HD-MTX (8 weeks)**  - 6MP 25 mg/m^2^ D1-56  - HD-MTX 5g/m^2^ D8, 22, 36, 50  - IT MTX Q2wk with HD- MTX (4x) | **HR Blocks 1, 2, 3**  The repeated HR Blocks 1, 2, 3 will follow the same method as above. |
| **Delayed Intensification** | **VDLD+CAM (6 weeks)**  *VDLD*  - Dexamethasone 8mg/m^2^ D1-21, taper over 9 days  - Doxorubicin 30mg/m^2^ D8, 15, 22, 29  -Vincristine 1.5mg/m^2^ D8,15, 22,29  - L-Asparaginase 5000units/m^2^ D8, 11, 14, 17,20,23  *CAM*  - Cyclophosphamide 1000mg/m^2^ D36  - Ara-C 75mg/m^2^ D38-41, D45-48  - 6MP 50 mg/m^2^ D36-49  - IT Triple D38 | **VDLD+CAM (6 weeks)**  *VDLD*  - Dexamethasone 8mg/m^2^ D1-21, taper over 9 days  - Doxorubicin 30mg/m^2^ D8, 15, 22, 29  -Vincristine 1.5mg/m^2^ D8,15, 22,29  - L-Asparaginase 5000units/m^2^ D8, 11, 14, 17,20,23  *CAM*  - Cyclophosphamide 1000mg/m2 D36  - Ara-C 75mg/m^2^ D38-41, D45-48  - 6MP 50mg/m^2^ D36-49  - IT Triple D38 |
| **Interim Maintenance** | **6-MP+MTX/VD (12 weeks)**  -6MP 50mg/ m^2^/day D1-84  -MTX 20mg/m^2^/week  -Dexamethasone 6mg/m^2^ on D43-49  -Vincristine 1.5mg/m^2^ D1, 8  - IT Triple D1 | / |
| **2nd Delay**  **Intensification** | **VDLA+VA +CAM**  *VDLA*  - Dexamethasone 6mg/m^2^ D1-7  -Vincristine 1.5mg/m^2^ D1,8  -HD-Ara-C 2g/m^2^ D2,4  - L-Asparaginase 25000units/m^2^ D4  - IT MTX D1  12 weeks usage of 6-MP+MTX/VD as Interim Maintenance.  *VA*  -teniposide 150mg/m^2^ D1,4,7  -Ara-C 300mg/m^2^ D1,4,7  Another 12 weeks usage of 6-MP+MTX/VD as Interim Maintenance.  *CAM*  - Cyclophosphamide 600mg/m^2^ D1  - Ara-C 75mg/m^2^ D3-6, D10-13  - 6MP 50mg/m^2^ D1-14 | / |
| **Maintenance** | **6-MP+MTX/VD (12 weeks) (up to 2-2.5 years from diagnosis)**  Patients will follow the same method as used in Interim Maintenance. | |

**Supplementary Table 2.** **CCLG-2008 chemotherapy protocol**

| **Treatment Block** | **Intermediate-risk** | **High-risk** |
| --- | --- | --- |
| **Induction** | **VDLD (5 weeks)**  - Prednisolone 60mg/m^2^ D1-7  - Dexamethasone 6mg/m^2^ D8-28, taper over 9 days  - L-Asparaginase 5000unit/m^2^ Day 8, 11, 14, 17, 20, 23, 26, 29  - Vincristine 1.5mg/m^2^ D8, 15, 22, 29  - Daunorubicin 25mg/m^2^ D8, 15, 22, 29(4x)  - IT MTX D1, Triple D15, 33 | **VDLD (5 weeks)**  - Prednisolone 60mg/m^2^ D1-7  - Dexamethasone 6mg/m^2^ D8-28, taper over 9 days  - L-Asparaginase 5000unit/m^2^ Day 8, 11, 14, 17, 20, 23, 26, 29 (8x)  - Vincristine 1.5mg/m^2^ D8, 15, 22, 29  - Daunorubicin 25mg/m^2^ D8, 15, 22, 29(4x)  - IT MTX D1, Triple D15, 33 |
| **Early intensification** | **CAM x 2 (week 5-9)**  - Cyclophophamide 1000mg/m^2^ D36  - Ara-C 75mg/m^2^ D38-41, 45-48  - 6MP 60mg/m^2^ D36-50  - IT Triple D38 | **CAM x 2 (week 5-9)**  - Cyclophophamide 1000mg/m^2^ D36  - Ara-C 75mg/m^2^ D38-41, 45-48  - 6MP 60mg/m^2^ D36-50  - IT Triple D38 |
| **Consolidation** | **6-MP+HD-MTX (8 weeks)**  - 6MP 25mg/m^2^ D1-56  - MTX 5gm/m^2^ D8, 22, 36, 50  - IT Triple Q2wk with HD-MTX (4x) | **HR Blocks 1, 2, 3, 1, 2, 3**  The HR Blocks 1, 2, 3,1,2,3 will follow the same method as BCH-2003. |
| **Delayed Intensification** | **VDLD+CAT (6 weeks)**  *VDLD*  - Dexamethasone 10 mg D1-7, D15-21 (no taper)  - Doxorubicin 25mg/m^2^ D1, 8, 15  - Vincristine 1.5mg/m^2^ D1, 8, 15  - L-Asparaginase 10000units/m^2^ D1, 4, 8, 11  *CAT*  - Cyclophophamide 1000mg/m^2^ D29  - Ara-C 75mg/m^2^ D31-34, D38-41  - 6TG 60 mg/m^2^ D29-42  - IT MTX D31, 38 | **VDLD+CAT (6 weeks)**  *VDLD*  - Dexamethasone 10 mg D1 -7, D15-21 (no taper)  - Doxorubicin 25 mg/m^2^ D 8, 15, 22, 29  - Vincristine 1.5 mg/m^2^ D 8, 15, 22, 29- L-Asparaginase 10000units/m^2^ D 8, 11, 15, 18 *CAT*  - Cyclophophamide 1000mg/m^2^ D36  - Ara-C 75mg/m^2^ D38-41, D45-48  - 6TG 60 mg/m^2^ D36-49  - IT Triple D38, 45 |
| **Interim Maintenance** | **6-MP+MTX (8 weeks)**  -6MP 50mg/m^2^/day D1-56  -MTX 20 mg/m^2^/week D1-56 | / |
| **2nd Delay Intensification** | **VDLD+CAT (6 weeks)**  *VDLD*  - Dexamethasone 10mg D1-7, D15-21 (no taper)  - Doxorubicin 25mg/m^2^ D1, 8, 15 (3x)  - Vincristine 1.5mg/m^2^ D1, 8, 15 (3x)  - L-Asparaginase 10000units/m^2^ D1, 4, 8, 11 (4x)  *CAT*  - Cyclophophamide 1000mg/m^2^ D29  - Ara-C 75mg/m^2^ D31-34, D38-41  - 6TG 60mg/m^2^ D29-42(14D)  - IT Triple D31, 38 | / |
| **Maintenance** | **6-MP+MTX /VD+IT (8 week cycle, male 11 cycles, female 8 cycles)**  -6MP 50mg/ m^2^/day D1-56  -MTX 20mg/m^2^/week D1-56  -Dexamethasone 6mg/m^2^ x 5 days every 4 weeks (D1-5, D29-33)  -Vincristine 1.5mg/m^2^ every 4 weeks (D1, 29)  -IT Triple Q8wk (d50) x 4 more doses (total 17 doses) | **6-MP+MTX/CA/VD+TIT (4 week cycle) (up to 2-2.5 years from diagnosis)**  -6MP 50mg/m^2^/day D1-14  -MTX 20mg/m^2^/week D1-14  -Cyclophosphamide 300mg/ m^2^ iv over I hour, Ara-C 300mg/ m^2^ iv over 1 hour. (From week 49 of maintenance, replaced by 6MP/MTX)  -Dexamethasone 6mg/m^2^/day po in bd or tid x 5 days,  -Vincristine 2mg/m^2^ iv on day 1 (maximal 2 mg). (from week 80 of maintenance, replaced by MTX/6MP)  -Triple IT Q4 weeks for 10 doses (week 3, on day of admission for CPM/Ara-C), total 23 doses. |

**Supplementary Table 3**. **Stratification criteria of BCH-2003 and CCLG-2008 treatment protocol**

| **Risk group** | **BCH-2003** | **CCLG-2008** |
| --- | --- | --- |
| **Intermediate risk** | -Good prednisone response  -No t(9;22)  -Bone marrow morphology was M1  at day 33  -Any one of:  Age≥6 or <1  WBC≥20×10^9^/L  T-cell  MLL rearrangements | -Good prednisone response  -Not(9;22) or MLL rearrangements  -Bone marrow morphology at day15 was M1/M2  -with IR protocol or M3 with SR protocol  -MRD<10^-2^ at day 33 and <10^-3^ at day 78  -Any one of:  Age<1 or age ≥10  WBC≥50×10^9^/L  T-cell  CNSL with no other high risk factor |
| **High risk** | Any one of:  -Poor prednisone response  -t(9;22)  -Bone marrow morphology was M2/M3 at day 33 | Any one of:  -Poor prednisone response  -t(9;22) or MLL rearrangements  -Bone marrow morphology was M2/M3 at day 33 and M3 on day 15 with IR protocol  -MRD≥10-^2^ at day 33 or MRD≥10^-3^ at week 12 |

**Supplementary Table 4. Clinical characteristics of patients according to protocols**

|  | **Protocol** | | ***P* value** |
| --- | --- | --- | --- |
|  | **BCH-2003 (%) CCLG-2008 (%)** | |  |
| **Total** | 27 (36.5) | 47 (63.5) |  |
| **Age(y)**  1-10  ≥10 | 16 (37.2)  11 (35.5) | 27 (62.8)  20 (64.5) | 0.879 |
| **Sex**  Male  Female | 24 (42.1)  3 (17.6) | 33 (57.9)  14 (82.4) | 0.066 |
| **WBC (10^9^/L)**  ＜100  ≥100 | 10 (34.5)  17 (37.8) | 19 (65.5)  28 (62.2) | 0.774 |
| **CNS involvement**  Present  Absent | 22 (32.4)  5 (83.3) | 46 (67.6)  1 (16.7) | 0.013 |
| **karyotype**  Normal  Structure abnormal  Numerical abnormal  Failure or Missing | 9 (29.0)  6 (35.3)  3 (42.9)  9 | 22 (71.0)  11 (64.7)  4 (57.1)  10 | 0.752 |
| **Mediastinal mass**  Present  Absent  Not known | 6 (21.4)  20 (48.8)  1 | 22 (78.6)  21 (51.2)  4 | 0.021 |
| **Prednisone response**  PGR  PPR  Not known | 6 (17.6)  10 (37.0)  11 | 28 (82.4)  17 (63.0)  2 | 0.087 |
| **D15 bone marrow**  M1  M2  M3  Missing | 16 (44.4)  4 (21.1)  1 (10.0)  6 | 20 (55.6)  15 (78.9)  9 (90.0)  3 | 0.055 |
| **Induction of remission**  CR  Failure  Not known | 26 (37.7)  1 (33.3) | 43 (62.3)  2 (66.7)  2 | 0.879 |
| **Risk group**  IR  HR  Not known | 7 (24.1)  18 (41.9)  2 | 22 (75.9)  25 (58.1) | 0.121 |

M1 (bone marrow blast ＜5%),M2 (bone marrow blast ≥5% and ＜25%), M3 (bone marrow blast ≥25%). WBC, white blood cell count; CNS, central nervous system; CR, complete remission; PGR, prednisone good responder; PPR, prednisone poor responder ; IR, intermediate risk; HR, high risk; SE, standard error.

^*^ By exact chi-square test

**Supplementary Table 5. Clinical characteristics of patients according to prednisone response.**

|  | **Prednisone response** | | ***P* value** |
| --- | --- | --- | --- |
|  | **PGR (%) PPR (%)** | |  |
| **Total** | 34 (55.7) | 27 (44.3) |  |
| **Age(y)**  1-10  ≥10 | 19 (54.3)  15 (57.7) | 16 (45.7)  11 (42.3) | 0.791 |
| **Sex**  Male  Female | 23 (52.3)  11 (64.7) | 21 (47.7)  6 (35.3) | 0.381 |
| **WBC (10^9^/L)**  ＜100  ≥100 | 18 (72.0)  16 (44.4) | 7 (28.0)  20 (55.6) | 0.033 |
| **CNS involvement**  Present  Absent | 1 (33.3)  33 (56.9) | 2 (66.7)  25 (43.1) | 0.423 |
| **karyotype**  Normal  Structure abnormal  Numerical abnormal  Failure or Missing | 16 (57.1)  9 (69.2)  2 (28.6)  7 | 12 (42.9)  4 (30.8)  5 (71.4)  6 | 0.215 |
| **SIl-TAL1 translocation**  Present  Absent  Missing | 6 (75.0)  21 (63.6)  7 | 2 (25.0)  12 (36.4)  13 | 0.543 |
| **MLL arrangement**  Present  Absent  Missing | 1 (33.3)  28 (62.2)  5 | 2 (66.7)  17 (37.8)  8 | 0.322 |
| **D15 Bone Marrow**  M1  M2  M3  Missing | 23 (74.2)  8 (44.4)  3 (30.0)  0 | 8 (25.8)  10 (55.6)  7 (70.0)  2 | 0.019 |
| **Induction of remission**  CR  Failure | 34 (59.6)  0 (0.0) | 23 (40.4)  4 (100.0) | 0.034 |

PGR, prednisone good responder; PPR, prednisone poor responder; WBC, white blood cell count; CNS, central nervous system; CR, complete remission; SE, standard error;

^*^ By exact chi-square test

**Supplementary Table 6. Clinical characteristics of patients according to bone marrow response on day 15.**

|  | **D15 Bone Marrow** | | ***P* value** |
| --- | --- | --- | --- |
|  | **M1/M2 (%) M3 (%)** | |  |
| **Total** | 55 (84.6) | 10 (15.4) |  |
| **Age(y)**  1-10  ≥10 | 33 (84.6)  22 (84.6) | 6 (15.4)  4 (15.4) | 1.0 |
| **Sex**  Male  Female | 41 (85.4)  14 (82.4) | 7 (14.6)  3 (17.6) | 0.764 |
| **WBC (10^9^/L)**  ＜100  ≥100 | 24 (92.3)  31 (79.5) | 2 (7.7)  8 (20.5) | 0.16 |
| **CNS involvement**  Present  Absent | 3 (100.0)  52 (83.9) | 0 (0.0)  10 (16.1) | 0.45 |
| **karyotype**  Normal  Structure abnormal  Numerical abnormal  Failure or Missing | 25 (89.3)  12 (85.7)  4 (57.1)  24 | 3 (10.7)  2 (14.3)  3 (42.9)  2 | 0.117 |
| **SIl-TAL1 translocation**  Present  Absent  Missing | 8 (100.0)  25 (78.1)  32 | 0 (0.0)  7 (21.9)  3 | 0.145 |
| **MLL arrangement**  Present  Absent  Missing | 2 (66.7)  37 (84.1)  26 | 1 (33.3)  7 (15.9)  2 | 0.437 |
| **Induction of remission**  CR  Failure  Not known | 53 (86.9)  1 (50.0)  1 | 8 (13.1)  1 (10.0)  1 | 0.142 |

M1 (bone marrow blast ＜5%),M2 (bone marrow blast ≥5% and ＜25%), M3 (bone marrow blast ≥25%). WBC, white blood cell count; CNS, central nervous system; CR, complete remission; SE, standard error.

^*^ By exact chi-square test

**Supplementary Table 7. Clinical characteristics of patients according to MRD risk group.**

|  | **MRD risk group** | | | **P value** |
| --- | --- | --- | --- | --- |
|  | **MRD-SR (%) MRD-IR (%) MRD-HR (%)** | | |  |
| **Total** | 9 (29.0) | 13 (42.0) | 9 (29.0) |  |
| **Age(y)**  1-10  ≥10 | 4 (20.0)  5 (45.5) | 9 (45.0)  4 (36.4) | 7 (35.0)  2 (18.1) | 0.301 |
| **Sex**  Male  Female | 6 (26.1)  3 (37.5) | 12 (52.2)  1 (12.5) | 5 (21.7)  4 (50) | 0.127 |
| **WBC (10^9^/L)**  ＜100  ≥100 | 3 (23.1)  6 (33.3) | 8 (61.5)  5 (27.8) | 2 (15.4)  7 (38.9) | 0.152 |
| **CNS involvement**  Present  Absent | 0 (0)  9 (30) | 1 (100)  12 (40) | 0 (0)  9 (30) | 0.489 |
| **SIl-TAL1 translocation**  Present  Absent  Missing | 3 (42.9)  5 (25.0)  1 | 3 (42.9)  8 (40.0)  2 | 1 (14.2)  7 (35.0)  1 | 0.517 |
| **MLL arrangement**  Present  Absent  Missing | 0 (0)  9 (31.0)  0 | 0 (0)  12 (41.4)  1 | 1 (100)  8 (27.6)  0 | 0.299 |
| **Prednisone response**  PGR  PPR  Missing | 6 (33.3)  3 (25) | 9 (50)  3 (25)  1 | 3 (16.7)  6 (50) | 0.138 |
| **D15 Bone Marrow**  M1/M2  M3  Missing | 8 (33.3)  1 (16.7) | 11 (45.8)  1 (16.7)  1 | 5 (20.9)  4 (66.7) | 0.09 |
| **Prednisone response**  PGR  PPR  Missing | **MRD-SR/MR**  15 (83.3)  6 (50)  1  19 (79.1)  2 (33.3)  1 | | 3 (16.7)  6 (50) | 0.102 |
| **D15 Bone Marrow**  M1/M2  M3  Missing |  |  | 5 (20.9)  4 (66.7) | 0.049 |

M1 (bone marrow blast ＜5%),M2 (bone marrow blast ≥5% and ＜25%), M3 (bone marrow blast ≥25%). WBC, white blood cell count; CNS, central nervous system; CR, complete remission; PGR, prednisone good responder; PPR, prednisone poor responder ; SE, standard error.

^*^ By exact chi-square test

**Supplementary Table 8. Clinical characteristics of patients with or without MRD data**

|  | **Patients with or without MRD** | | **P value** |
| --- | --- | --- | --- |
|  | **With MRD (%) Without MRD (%)** | |  |
| **Total** | 36 (48.6) | 38 (51.4) |  |
| **Age(y)**  1-10  ≥10 | 22 (51.2)  14 (45.2) | 21 (48.8)  17 (54.8) | 0.61 |
| **Sex**  Male  Female | 26 (45.6)  10 (58.8) | 31 (54.4)  7 (41.2) | 0.339 |
| **WBC (10^9^/L)**  ＜100  ≥100 | 16 (55.2)  20 (44.4) | 13 (44.8)  25 (55.6) | 0.367 |
| **CNS involvement**  Present  Absent | 1 (16.7)  35 (51.5) | 5 (83.3)  33 (48.5) | 0.102 |
| **Mediastinal mass**  Present  Absent  Not known | 18 (64.3)  15 (36.6)  3 | 10 (35.7)  26 (63.4)  2 | 0.024 |
| **karyotype**  Normal  Structure abnormal  Numerical abnormal  Failure or Missing | 16 (51.6)  10 (58.8)  4 (57.1)  6 | 15 48.4)  7 (41.2)  3 (42.9)  13 | 0.882 |
| **SIl-TAL1 translocation**  Present  Absent  Missing | 7 (87.5)  24 (70.6)  5 | 1 (12.5)  10 (29.4)  27 | 0.328 |
| **MLL arrangement**  Present  Absent  Missing | 1 (33.3)  32 (69.6)  3 | 2 (66.7)  14 (30.4)  22 | 0.195 |
| **Prednisone response**  PGR  PPR  Not known | 23 (67.6)  12 (44.4)  1 | 11 (32.4)  15 (55.6)  12 | 0.069 |
| **D15 Bone Marrow**  M1  M2  M3  Missing | 15(41.7)  13(68.4)  7 (70.0)  1 | 21(58.3)  6 (31.6)  3 (30.0)  8 | 0.09 |
| **Risk group**  IR  HR  Not known | 18 (62.1)  18 (41.9) | 11 (37.9)  25 (58.1)  2 | 0.093 |
| **Induction of remission**  CR  Failure | 35 (50.7)  1 (20.0) | 34 (49.3)  4 (80.0) | 0.184 |

M1 (bone marrow blast ＜5%),M2 (bone marrow blast ≥5% and ＜25%), M3 (bone marrow blast ≥25%). WBC, white blood cell count; CNS, central nervous system; CR, complete remission; PGR, prednisone good responder; PPR, prednisone poor responder ; IR, intermediate risk; HR, high risk; SE, standard error.

^*^ By exact chi-square test
